# Evidence for the communicative function of human-directed gazing in 6- to 7-week-old dog puppies

**DOI:** 10.1007/s10071-024-01898-y

**Published:** 2024-09-23

**Authors:** Stefanie Riemer, Alina Bonorand, Lisa Stolzlechner

**Affiliations:** 1 Messerli Research Institute, Department of Interdisciplinary Life Sciences, Vetmeduni Vienna, Vienna, 1210 Austria; 2 Division of Animal Welfare, Vetsuisse Faculty, University of Bern, Bern, 3012 Switzerland; 3 Department of Behavioral & Cognitive Biology, University of Vienna, Vienna, 1030 Austria

**Keywords:** Gaze alternation, Referential looking, Ontogeny, Dog-human communication, Eye contact, Social referencing

## Abstract

In human infants, the ability to show gaze alternations between an object of interest and another individual is considered fundamental to the development of complex social-cognitive abilities. Here we show that well-socialised dog puppies show gaze alternations in two contexts at an early age, 6–7 weeks. Thus, 69.4% of puppies in a novel object test and 45.59% of puppies during an unsolvable task alternated their gaze at least once between a person’s face and the object. In both contexts, the frequency of gaze alternations was positively correlated with the duration of whimpering, supporting the communicative nature of puppies’ gazing. Furthermore, the number of gaze alternations in the two contexts was correlated, indicating an underlying propensity for gazing at humans despite likely different motivations in the two contexts. Similar to humans, and unlike great apes or wolves, domestic dogs show gaze alternations from an early age if they are well-socialised. They appear to have a genetic preparedness to communicate with humans via gaze alternations early in ontogeny, but they may need close contact with humans for this ability to emerge, highlighting the interactive effects of domestication and environmental factors on behavioural development in dogs.

**Supplementary Information:**

The online version contains supplementary material available at 10.1007/s10071-024-01898-y.

## Introduction

The emergence of gaze alternations between an object of interest and another individual is considered to be fundamental to the development of complex social-cognitive abilities in infants, from theory of mind to language (Lucca et al. 2018). In humans, the ability to gaze alternate emerges early in life, at approximately 8–10 months (Beuker et al. 2013; Carpenter et al. 1998; Lucca et al. 2018). In contrast, in great apes, spontaneous gaze alternations without prior ostensive cueing appear to emerge much later. Lucca et al. (2018) tested sanctuary-housed chimpanzees in a food-requesting task with a human experimenter. The younger subjects aged 3–6 years rarely alternated the gaze between the task and the person. Only in the older age group of 6–9 years did gaze alternations increase considerably and thus at a much later developmental stage than is typical in humans (Lucca et al. 2018). In the same study, although a relatively lenient criterion for gaze alternation was used (gazing at both the object and a person within a large time interval of up to five seconds), bonobos of all ages rarely showed gaze alternations at all, possibly reflecting their slower cognitive development compared to chimpanzees (Lucca et al. 2018). In a population of wild chimpanzees, the youngest individual to show (triadic) gaze alternations between a threatening object and a conspecific was 25 months old (Dezecache et al. 2019). Thus, it was suggested that what sets humans apart from other great apes is not necessarily the *production* of gaze alternations, but rather the *early* production of gaze alternations (Lucca et al. 2018).

Domestic dogs are renowned for their readiness to take up eye contact and show gaze alternations directed at humans (Kaminski et al. 2012; Merola et al. 2012a, b; Miklósi et al. 2003; Prato-Previde and Marshall-Pescini 2014; Téglás et al. 2012). Their high sensitivity to human communication from an early age is suggested to be a hallmark of domestication (Bray et al. 2021; Byosiere et al. 2023; Hare et al. 2002, 2010; Riedel et al. 2008; Salomons et al. 2021; Virányi et al. 2008), and this appears to be related to their readiness to make eye contact (Virányi et al. 2008). While much evidence demonstrates dog puppies’ ability to respond to human social cues from a young age (Bray et al. 2021; Byosiere et al. 2023; Gácsi et al. 2009; Miklósi et al. 2003; Riedel et al. 2008; Rossano et al. 2014; Salomons et al. 2021), there is a relative lack of studies on the ontogeny of social communication initiated by the puppies.

Human-directed gazing by dogs is investigated most often in one of two contexts: (1) when a desired reward is out of reach or otherwise unattainable for the dog, especially in the so-called ‘unsolvable task’ paradigm (e.g. Carballo et al. 2020; Cavalli et al. 2020; Lazarowski et al. 2020; Marshall-Pescini et al. 2009, 2013; Mendes et al. 2021a; Sanford et al. 2018; Scandurra et al. 2015) or (2) when the dog is exposed to an unfamiliar object or human (which/who might potentially be suspicious, Duranton et al. 2016, 2017; Fugazza et al. 2018; Merola et al. 2012a, b, 2013; Yong and Ruffman 2015).

When dogs are unable to access a reward, such as in the ‘unsolvable task’, they will soon look back at the human (e.g. Gaunet 2008, 2010; Gaunet and Deputte 2011; Mendes et al. 2021a; Miklósi et al. 2000; Turcsán et al. 2018). They do this not only by establishing direct eye contact with the person, but also by alternating their gaze between the human and the problem. Gaze alternations are commonly defined as incorporating a two-step sequence in which the subject first looks at the stimulus and then towards a person – or vice versa – within a short period of time, typically 2 s (e.g. Fugazza et al. 2018; Gaunet 2008, 2010; Gaunet and Deputte 2011; Hirschi et al. 2022; Mendes et al. 2021a; Miklósi et al. 2000; Nawroth et al. 2016; Savalli et al. 2014). Alternating the gaze is suggested as evidence of an intentional and directional communicative act (Gaunet and Deputte 2011) and is also referred to as ‘referential looking’ (but see a critical review on inferring intentionality in Mocha and Burkart 2021).

There is evidence that dogs’ gazing at a person’s face can serve to request help to attain a reward (Hirschi et al. 2022), and since dogs can successfully modify people’s behaviour by looking into their faces, it has even been suggested that this behaviour can be interpreted as “social tool use” (Kubinyi et al. 2007). Moreover, dogs show audience effects and further attention-getting behaviours such as vocalising or touching a person, all indicating communicative intent (e.g. Gaunet 2008; Gaunet and Deputte 2011; Hirschi et al. 2022; Marshall-Pescini et al. 2009, 2013; Miklósi et al. 2000; Savalli et al. 2014).

Besides situations in which dogs are faced with an unsolvable problem, gaze alternations are also shown in situations of uncertainty, such as in the presence of a novel object or person (Fugazza et al. 2018; Merola et al. 2012a, b, 2013; Yong and Ruffman 2015). In such situations, dogs not only observe humans’ reactions to the stimulus, they also adjust their behaviour to the human’s emotional reaction, i.e., they show social referencing (Fugazza et al. 2018; Merola et al. 2012a, b, 2013; Yong and Ruffman 2015). In this case, alternating the gaze between an object of interest and a person appears to serve as an information seeking strategy (Fugazza et al. 2018; Roberts et al. 2008).

Despite the wealth of studies on adult dogs’ gaze behaviour towards humans (e.g. reviewed in Cavalli et al. 2018; Mendes et al. 2021a) as well as several studies on the ontogeny of their ability to read human communicative signals (e.g. Byosiere et al. 2023; Bray et al. 2021; Hare et al. 2002, 2010; Riedel et al. 2008; Salomons et al. 2021; Virányi et al. 2008), few studies have investigated the ontogeny of human-directed gazing in dogs, and in particular, if and when such gazing may serve a communicative function, rather than dogs ‘just looking around’ in the environment (cf. Cimarelli and Range 2022).

In Passalacqua et al. (2011), approximately half of the two-month-old puppies gazed at the experimenter during an unsolvable task, but gaze alternations were rare, being only shown by seven of 97 puppies. In Lazarowski et al. (2019), candidate detection dogs tested in an unsolvable task paradigm at 3, 6 and 11 months rarely gazed at people until 11 months of age, and the authors concluded that – as in nonhuman primates – gaze alternation emerges at a later developmental stage than in our own species.

In contrast to these findings, in a social referencing paradigm, nearly all puppies (aged eight weeks) alternated their gaze between a novel object and the experimenter (Fugazza et al. 2018). Moreover, they also adjusted their behaviour towards the object based on the emotional cue given by the person, similar as human infants, thus fulfilling the criteria of social referencing (Fugazza et al. 2018). The reason for the differences between these studies in the occurrence of gaze alternations in young puppies could lie in the type of task (unsolvable tasks vs. novel object) or in different prior socialisation experiences.

While there is some preliminary evidence that individual differences in social gazing behaviour in dogs may be linked to personality as in humans (Prato-Previde and Marshall-Pescini 2014), no study to date has compared the effect of these different contexts (novel object vs. unsolvable task) on gazing behaviour in dogs.

Here we investigated gaze alternations in 83 well-socialised puppies of various breeds, aged 6–7 weeks. Gazing and duration of whimpering were coded in two different contexts: (a) an unsolvable task paradigm and (b) exposure to a novel object.

We predicted that young puppies show gaze alternations in both contexts and that the frequency of gaze alternations is correlated across situations. As an indication of the communicative function of gaze alternations (cf. Prato-Previde and Marshall-Pescini 2014), we predicted a positive association between the frequency of gaze alternations and the duration of whimpering within subtests.

## Methods

### Subjects

Eighty-three dog puppies (*Canis familiaris*) of eight different breeds from 12 litters were included in the study (Supplementary Table 1). Forty-eight subjects were female and 35 were male. All participating breeders (*N* = 11, one breeder participated with two litters) were small-scale breeders and most belonged to the FCI (Féderation Cynologique Internationale). The puppies spent most of their time in the house. The breeders interacted with them several times daily beyond feeding and cleaning, and additionally, the puppies were part of a study exploring the effect of early enrichment on behaviour in dogs (Stolzlechner et al. 2022). To this end, the experimenter (LS) visited all puppies twelve times over four weeks between the ages of 3 and 5–6 weeks. Counterbalanced within litters, half the puppies were presented with novel objects, problem-solving tasks, and exposed to potentially startling stimuli. This involved some handling (e.g. to place the puppy at the starting point of the problem-solving task) as well. LS spent the same amount of time with the control group, cuddled or played with these puppies, performed the same type of handling and gave them the same amount of food as the experimental group received on the corresponding day (Stolzlechner et al. 2022). Thus, all puppies had rich social experiences, but less human contact than the hand-raised dogs and wolves from comparative studies that spent the first weeks of life (since before eye-opening) with their hand raisers, 24 h a day (Gácsi et al. 2005; Virányi et al. 2008).

### Behavioural testing

At the age of 41 to 52 days (mean 43.4 days ± SD 3.1 days), the puppies were tested individually in a behaviour test adapted from Riemer et al. (2013, 2014). Due to the risk of disease contraction for the young puppies, all tests were carried out at the breeders’ homes, in a room that was unfamiliar to the puppies. The test lasted approximately 20 min and consisted of six subtests: exploration of an unfamiliar room, interaction with a friendly stranger, a problem-solving task that was rendered unsolvable in the second trial, a startle test (loud noise), and a novel object test (see Stolzlechner et al. 2022; for details). Of these, only the unsolvable problem test and the novel object test were analysed for the current manuscript.

During the novel object test (duration 2 min), a battery-powered cat toy that looked like a colourful paper bag (approximately 20 × 10 × 5 cm) and moved on the spot was placed at a distance of approximately 1.5 m from the puppy. The puppy was free to move and explore.

The problem-solving task consisted of two parts. During the solvable problem task, the experimenter placed a few pieces of food under a cup in full view of the puppy, which the puppy could access by knocking over the cup. In the second part immediately thereafter, the unsolvable task (duration 2 min), the same procedure was followed except that the cup was attached to the surface so that it could no longer be knocked over, rendering the food inaccessible.

Three people (all female) of varying acquaintance with the puppies were present during the tests: the breeder, the experimenter (LS, who had visited all puppies twelve times for the enrichment study, Stolzlechner et al. 2022), and the camerawoman, who filmed the entire test with a handheld camera (unfamiliar to the puppies except when playing a friendly stranger in subtest 2, ‘greeting’). The three people present were observing the puppy but did not interact with her/him during the subtests relevant for the current manuscript.

The order of subtests was the same for all puppies, since the focus of Stolzlechner et al. (2022) was on individual behavioural differences. Similarly, if gazing was affected by the preceding subtests, all puppies would have the same prior experiences. We see no reason to suspect that the first two subtests (room exploration and interaction with the unfamiliar person) affected gazing behaviour in the unsolvable task relative to other studies, since in most cognitive studies, the experiment is performed following room habituation and familiarisation with the experimenter. However, we cannot rule out that the startling experience of the loud noise prior to the novel object test increased human-directed gazing.

### Coding and analysis

Videos were coded by AB in Solomon Coder (© András Péter, www.solomoncoder.com). The following variables were of interest for the current study: frequency of gaze alternations and duration of whimpering during the problem-solving task and the novel object test. A gaze alternation was defined after Fugazza et al. (2018); Gaunet 2008), 2010); Gaunet and Deputte (2011); Hirschi et al. (2022); Lakatos et al. (2012); Nawroth et al. (2016); Mendes et al. (2021a, b); Miklósi et al. (2000), 2005) as either shifting the gaze from the stimulus (problem-solving task/ novel object) to a person or from a person to the stimulus within two seconds. Thus, to be coded as a gaze alternation, the puppy’s gaze (inferred from the direction of the face) had to be directed at both the object and a person’s face within a timeframe of two seconds. We only counted gazes that were directed at a person’s face, not at the rest of the body, and inferred gazes to a person’s face by drawing a mental line between the puppy’s eyes and the human’s face.

Although we differentiated between persons during coding, the absolute frequency of gaze alternations per person was low, and therefore all gaze alternations were summarised for the problem-solving task and the novel object task, respectively. Whimpering (producing a high-pitched noise) was measured as a duration in both subtests.

Having found a much higher prevalence of gaze alternations in the unsolvable task than a previous study, we additionally calculated the latency to the first gaze alternation in order to determine whether this result could be explained by the longer test duration (2 min vs. 1 min), or whether it was more likely to be attributed to other differences between the populations.

Reliability coding for the frequency of gaze alternations was performed for 12 puppies (one randomly selected puppy per litter) by an additional rater not involved in the study. Reliability coding for the duration of whimpering was performed for one randomly selected puppy per litter by LS as published in Stolzlechner et al. (2022). Cronbach’s alpha was above 0.82 for all variables (Supplementary Table 2).

Statistical analyses were carried out using R Version 3.6.1. As the data were non-normally distributed, nonparametric statistics were used. Figures were prepared in Statistica 6.1.

If puppies were out of sight or gazing could not be coded for other reasons for more than 10 s, the number of gaze alternations from this subtest was designated as NA. Additionally, we excluded puppies from the analysis of the unsolvable task if they did not succeed in solving the solvable task (*N* = 15). Thus, gazing data were available from 72 puppies for the novel object test and from 69 puppies for the unsolvable task.

The two treatment groups from Stolzlechner et al. (2022) did not differ significantly in the frequency of gaze alternations during the novel object test (Wilcoxon rank sum test, W = 800, *p =* 0.076) or the unsolvable task (Wilcoxon rank sum test, W = 588, *p* = 0.3907); therefore data from all puppies were pooled for the analysis.

Given the non-normal distribution of the data, Spearman rank correlation tests were performed in order to assess the relationship between the frequency of gaze alternations and the duration of whimpering in each subtest. Further Spearman rank correlation tests were used to correlate the frequency of gaze alternations during the unsolvable task with the frequency of gaze alternations during the novel object test and to test for an association between puppies’ age and the frequency of gaze alternations in each test.

When applying Bonferroni correction for the performance of five correlational tests, results with a *p*-value < 0.01 can be considered significant. As all significant results were below this corrected threshold, the original *p*-values are reported in the Results.

## Results

In the novel object test, 50 of the 72 subjects where gazing could be coded (69.4%) exhibited at least one gaze alternation. Of these, 39 puppies (54.17%) alternated their gaze within the first minute of the test. The median latency to the first gaze alternation was 17.2 s (IQR: 10.5–22.8 s). In the unsolvable task, 31 of the 68 puppies that had successfully acquired the food in the solvable task (45.59%) showed at least one gaze alternation. Twenty of these puppies (29.4%) alternated their gaze within the first minute of the test. The median latency to the first gaze alternation was 50.8 s (IQR: 26.4–83 s). No correlation between the frequency of gaze alternations and age was found (novel object test: r_S_=0.12, *p =* 0.329; unsolvable problem task: r_S_=0.05, *p =* 0.701).

### Gaze alternations are correlated with whimpering

**Fig. 1:**
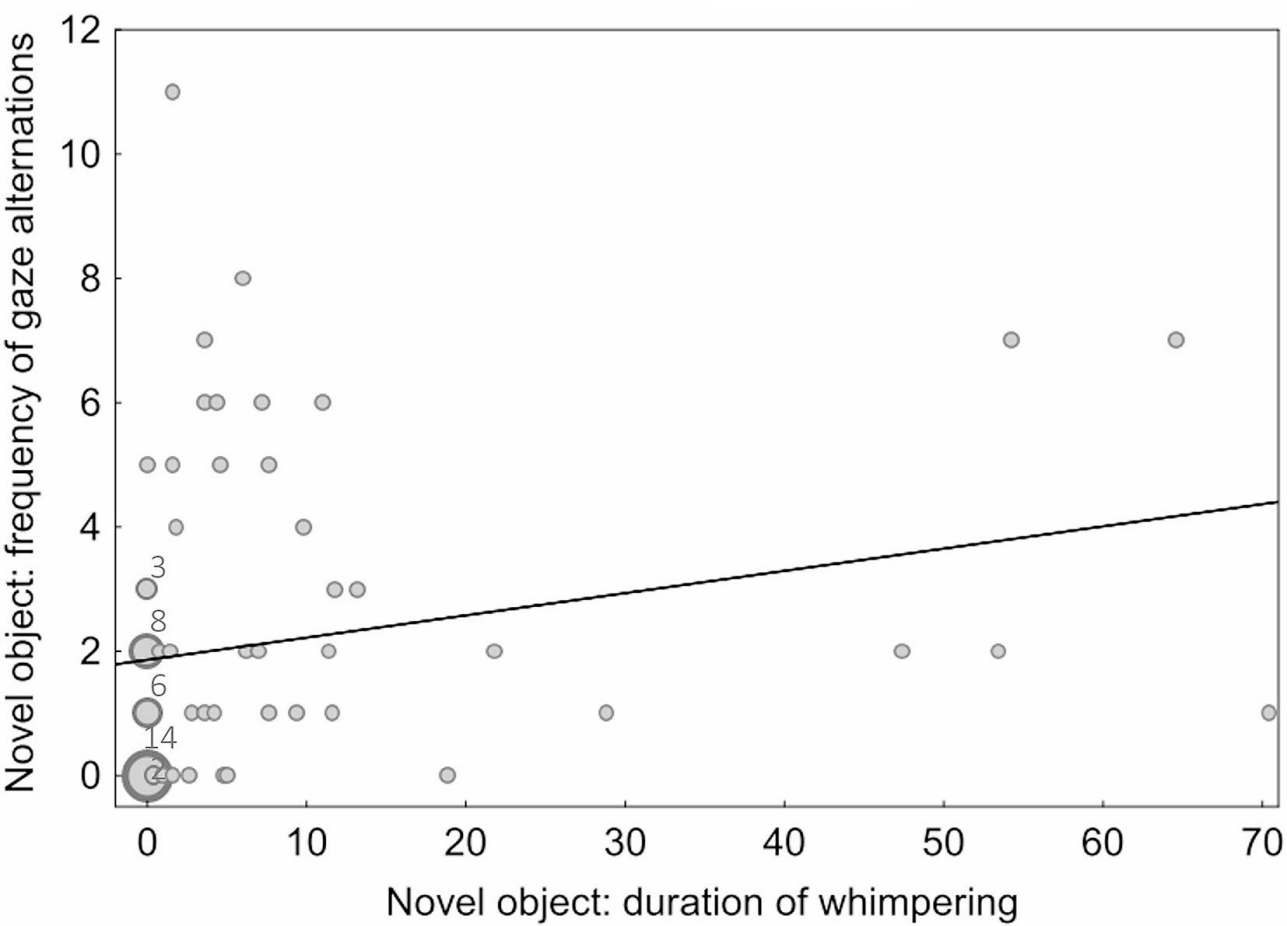
Frequency scatter plot depicting the number of gaze alternations against the duration of whimpering (s) during the novel object test. Each bubble represents one or several data points. The smallest bubbles correspond to one subject, bigger bubbles correspond to a higher number of subjects with identical data (e.g. for 14 puppies both the duration of whimpering and the frequency of gaze alternations during the novel object test was 0). The numbers above the bubbles indicate how many puppies showed the corresponding frequency of gaze alternations

**Fig. 2:**
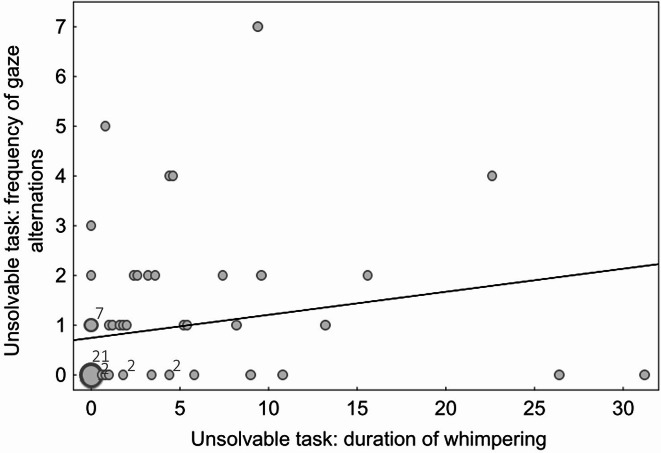
Frequency scatter plot depicting the number of gaze alternations against the duration of whimpering (s) during the unsolvable task. Each bubble represents one or several data points. The smallest bubbles correspond to one subject, bigger bubbles correspond to a higher number of subjects with identical data (e.g. for 21 puppies both the duration of whimpering and the frequency of gaze alternations during the unsolvable task was 0). The numbers above the bubbles indicate how many puppies showed the corresponding frequency of gaze alternations

There was a significant moderate positive correlation between the frequency of gaze alternations and whimpering in both the novel object test (r_S_=0.38 *p =* 0.001, Fig. 1) and the unsolvable task (r_S_=0.36, *p =* 0.003, Fig. 2).

### Gaze alternations are correlated across contexts

**Fig. 3:**
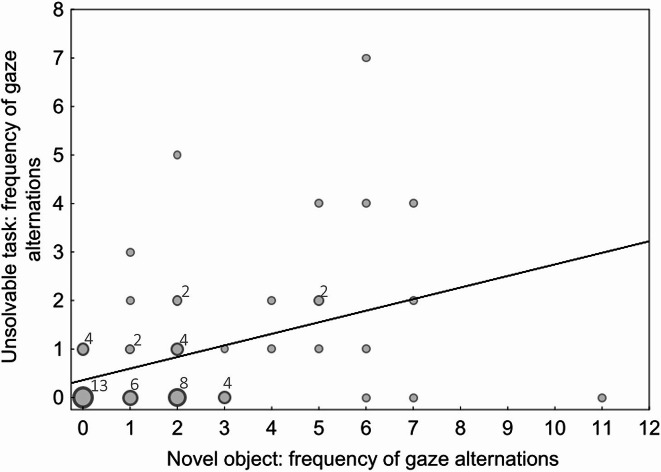
Frequency scatter plot depicting the number of gaze alternations during the unsolvable task against the number of gaze alternations during the novel object test. Each bubble represents one or several data points. The smallest bubbles correspond to one subject, bigger bubbles correspond to a higher number of subjects that had identical gazing data (e.g. 13 puppies never showed a gaze alternation during either the novel object test or the unsolvable task). The numbers above the bubbles indicate how many puppies showed the corresponding frequency of gaze alternations

A significant moderate positive correlation (r_S_=0.39, *p =* 0.0018) was observed between the frequency of gaze alternations in the novel object test and in the unsolvable task (Fig. 3).

## Discussion

We show that a high proportion of well-socialised dog puppies exhibit gaze alternations towards people in two different contexts already in the early socialisation period, aged six to seven weeks. Almost half of the subjects showed one or more gaze alternations during the unsolvable task, and more than two-thirds showed at least one gaze alternation during the novel object test.

Alternative explanations for human-directed gazing have been proposed, such as randomly looking around and/or gazing at salient objects in the environment (Cimarelli and Range 2022; Lazzaroni et al. 2020). However, there are several lines of evidence that puppies’ human-directed gazing served a social-communicative function. We coded gaze alternations, rather than just gazing at a person. This is relevant because alternating the gaze between a person and a relevant object in the environment can be an indicator of intentionality and the likely referential nature of the gazing behaviour (reviewed in Prato-Previde and Marshall-Pescini 2014). A significant correlation between the frequency of gaze alternations and the duration of whimpering (communicative signals that could potentially facilitate social interactions, Gácsi et al. 2005) in both studied contexts further supports the communicative function of the observed gaze alternations. Additionally, we only coded gaze directed at a person’s face, but not at other parts of the body to exclude “random” gazing in a person’s direction as much as possible. Thus, gazing at a person’s face usually required puppies to lift up their heads. Interestingly, in humans, younger children will gaze at any part of their parent’s body, but from 10 to 13 months of age, they will gaze preferentially at the face (Walden and Ogan 1988).

In human infants, “checking behaviour” (defined as looking at an adult without the intention to share and/or not integrating an object and the other in one interaction) is typically the first communicative skill to emerge, being present by eight or nine months (Beuker et al. 2013). (Triadic) gaze alternations (i.e. gazing from an adult to an object and back to the adult or vice versa, commonly referred to as “sharing attention”, but see critiques of this interpretation e.g. in Carpenter and Call 2013) have been documented as emerging between eight and ten months (Beuker et al. 2013; Carpenter et al. 1998; Carpenter and Call 2013).

It is unfortunate that there is inconsistency in the usage of “gaze alternation” across studies. Some human studies (e.g. Marshall-Pescini et al. 2013; Nyström et al. 2019; Thorup et al. 2018), great ape studies (e.g. Lucca et al. 2018), and most dog studies (Fugazza et al. 2018; Gaunet 2008, 2010; Gaunet and Deputte 2011; Hirschi et al. 2022; Lakatos et al. 2012; Mendes et al. 2021a, b; Miklósi et al. 2000, 2005; Savalli et al. 2014) used 2-way gaze alternations (gazing either from the object to the human or from the human to the object).

While a study measuring 2-way gaze alternations in adult dogs and children aged 15–27 months during an unsolvable task found that gaze alternations were shown by 73% of the dogs and 77% of the children (Marshall-Pescini et al. 2013), some of the classic infant studies employed the criterion of 3-way gaze alternations (object – caregiver – object)(Beuker et al. 2013; Carpenter et al. 1998; Lord et al. 2000). Thus direct comparisons with most dog studies are not entirely possible (but see Merola et al. 2012a, b, 2013). Nonetheless, even simple gazes (referred to as “checking behaviour”) were found to be present in all infants tested by eight to nine months of age (Beuker et al. 2013).

Thus, it is of interest that a relatively high proportion of puppies showed 2-way gaze alternations already at six to seven weeks of age. From an epigenetic perspective (based on an analysis of methylation in the genome with aging), an age of eight weeks in a common dog breed, the Labrador retriever, was found to correspond to approximately nine months in humans (Wang et al. 2020). This suggests that gaze alternations involving a human partner may emerge at a similar relative age in companion dog puppies as in our own species, at least if they are well socialised to humans.

It has been highlighted that what differentiates humans from our close relatives are not only more advanced social-cognitive skills, but in particular their early emergence in ontogeny (reviewed in Lucca et al. 2018). This is suggested to be a prerequisite for other sophisticated cognitive abilities including language and culture (see also an association between early gaze following and language development, Beuker et al. 2013). Remarkably, when it comes to the emergence of communicative gazing (at humans), humans and dogs appear to be more similar to each other than they are to either our closest relatives (great apes) or to dogs’ closest relatives (wolves).

One study investigated the ontogeny of (two-way) gaze alternations in the context of an unsolvable task in bonobos and chimpanzees from sanctuaries. Most of the subjects were victims of the wildlife trade and had been fostered in human families from a young age. Even as adults, the bonobos in this study rarely showed gaze alternations. In the chimpanzees, gaze alternations were commonly shown in communicative contexts, but were rare until six years of age, increasing greatly only between six and nine years (Lucca et al. 2018) and thus clearly later in ontogeny than in human infants and dog puppies. A small study on wild chimpanzees indicates that (three-way) gaze alternations between a conspecific and an unfamiliar object (a fake spider) were shown from 25 months of age (Dezecache et al. 2019).

Based on performance in cognitive batteries, previous comparative research demonstrated parallels in the socio-cognitive structure of (adult) dogs and human infants, which set them apart from age-matched chimpanzees or bonobos. These parallels were suggested to have been acquired through convergent evolution (MacLean et al. 2017). Our study adds to this finding by demonstrating that dog-human parallels in communicative skills are not limited to adult dogs (as in MacLean et al. 2017), but that ontogenetic pathways for some social-cognitive abilities appear to be similar in both species. While several previous studies documented dogs’ ability to follow human social cues from a young age (e.g. Bray et al. 2021; Salomons et al. 2021), the current study demonstrates that companion dogs can also initiate communicative interactions with humans early in ontogeny in a manner that might be considered referential.

Regarding comparisons with dogs’ closest extant relatives, wolves, studies have consistently demonstrated that domestic dogs show more human-directed gazing than wolves of the same age when individuals of both species were reared and socialised in an identical manner (Gácsi et al. 2005, 2009; Virányi et al. 2008), or even when wolves were raised with much more human contact than the dogs (Salomons et al. 2021).

While higher problem-solving persistence in wolves might explain this difference in the unsolvable task paradigm (Marshall-Pescini et al. 2017), similar differences were also found in other contexts. For instance, in Gácsi et al. (2005), 5-week-old dogs gazed more at a human’s face than same-aged wolves in situations with a passive human who was paired with different other stimuli. In a study on action matching, mother-reared dog puppies spent more time watching a human demonstrator than both hand-raised wolf pups and mother-reared kittens (Fugazza et al. 2023).

Furthermore, in a pointing experiment, wolves at three ages (8 weeks, 3–4 months and as adults) had a higher latency to make eye contact with the experimenter than dogs of the equivalent ages, even though all subjects had been hand-raised and reared identically until at least 3–4 months of age (Gácsi et al. 2009). In another pointing study, hand-raised wolves had a higher latency to take up eye contact with humans at four months compared to both hand-raised and mother-raised companion dogs. By 11 months, however, the wolves reached a similar level of establishing and maintaining eye contact with the pointing experimenter as the dogs, probably as a result of the extensive training and socialisation. At the same time, their ability to follow the more difficult momentary distal pointing reached a similar level as that of (untrained) companion dogs. The authors suggest that acquiring readiness to take up eye contact with the experimenter was key to success in following the pointing (Virányi et al. 2008).

Thus, it is clear that differences in the socio-cognitive development exist between dogs and wolves even when the two species are raised in an identical manner (Gácsi et al. 2005, 2009; Virányi et al. 2008), or when the wolves have more social experiences with humans than the dog puppies (Salomons et al. 2021). Whether dogs’ greater propensity to gaze at humans and to follow pointing gestures from an early age is due to direct effects of domestication on dogs’ social-cognitive ability (Hare et al. 2002; Miklósi et al. 2004; Salomons et al. 2023), whether wolves’ lower performance results because they are less likely to accept a human partner as a social partner (cf. Gácsi et al. 2009; Udell et al. 2010) and/ or have lower fear and aggression thresholds towards humans (Gácsi et al. 2005; Hansen Wheat et al. 2023; Hare and Tomasello 2005), and to what extent dog-wolf differences are the product of genes vs. environmental influences is still a matter of debate (see some competing hypotheses in Gácsi et al. 2009; Hansen Wheat et al. 2023; Range and Virányi 2015; Salomons et al. 2023; Udell et al. 2010). Some scholars highlight the interactive effects of evolutionary and environmental processes during ontogeny on dogs’ preparedness to attend to humans’ faces and sensitivity for salient human communicative cues (Gácsi et al. 2009; Udell et al. 2010; Udell and Wynne 2010).

Indeed, experience with humans seems to be important for communicative gazing also in dogs. For instance, compared to free-ranging dogs, companion dogs gazed longer at a human during an unsolvable task (Lazzaroni et al. 2020) and in a training for eye contact task (Brubaker et al. 2019). In contrast, no difference between owned companion dogs and shelter dogs emerged in the latter study (Brubaker et al. 2019).

Lazarowski et al. (2019) repeatedly tested a cohort of prospective detection dogs. In this population, human-directed gazing was nearly absent at three and six months of age and did not increase significantly until 11 months. Lazarowski et al. (2019) suggested that this late emergence of gazing might be explained by kennel-rearing of the subjects. Kennel dogs have fewer human interactions and thus less opportunity to learn about communicating with humans (Lazarowski et al. 2019). In line with this, adult Labrador retrievers kept in kennels looked back later and spent less time gazing towards people during an unsolvable task than dogs of the same breed that lived indoors with their owners (D’Aniello and Scandurra 2016).

Lazarowski et al. (2019) also suggest that more learning opportunities in the interaction with humans could explain why puppies’ gazing increased from two to four months of age in Passalacqua et al. (2011). Interestingly, in the current study, gaze alternations were much more common in our six- to seven-week-old puppies than in the eight-week-old puppies in Passalacqua et al. (2011). In our sample, 45.59% showed at least one gaze alternations during the unsolvable task, compared to only 7% in Passalacqua et al. (2011), although approximately half did gaze at the experimenter without alternating the gaze.

This could possibly be explained by some procedural differences between the studies. We only had one solvable trial, whereas Passalacqua et al. (2011) had three, which may have increased puppies’ persistence due to the higher number of previous successes, and thus reducing the probability of looking back (cf. Marshall-Pescini et al. 2017). Our test time was longer (two minutes) than in Passalacqua et al. (2011)(one minute), which is especially relevant if dogs only start gazing back after they tried to solve the problem on their own for a while. However, the analysis of latency to first gaze showed that 29.4% of puppies in our study exhibited a gaze alternation within the first minute of the test. Thus, the differences between studies cannot be explained by test duration only. Finally, the puppies in our study had more opportunity to gaze at people because three people were present compared to only the unfamiliar experimenter in Passalacqua et al. (2011).

Regarding possible breed effects, 80% of our sample belonged to hunting or herding breeds (compared to 48% in Passalacqua et al. (2011), which are considered to be highly cooperative and may have a higher propensity for human-directed gazing. For instance in Passalacqua et al. (2011), gazing was more common in hunting/herding breeds than in other breed groups. Nonetheless, this difference was not yet present in the youngest age group at eight weeks and emerged only at 4.5 months (Passalacqua et al. 2011). It is thus unlikely that the extent of the difference between the studies can be explained by breed effects alone.

Perhaps most importantly, the puppies in the current studies were raised indoors, had experienced extensive socialisation and received extra attention by the experimenter (LS) on 12 separate days for the enrichment study (Stolzlechner et al. 2022). In contrast, the puppies in Passalacqua et al. (2011) had fewer socialisation experiences – they were raised in pens and had no more than four daily interactions with people. The intensive contact with humans, both within and outside the breeder family, may be key to the puppies in our study being more communicative towards people than the puppies in in Passalacqua et al. (2011) and the juvenile dogs in Lazarowski et al. (2019).

In contrast to the eight-week-old puppies faced with an unsolvable task in Passalacqua et al. (2011), almost all the puppies of a similar age showed at least one gaze alternation in a social referencing paradigm in Fugazza et al. (2018). 95% of the puppies alternated their gaze between a novel object and a neutral experimenter, and all puppies did so when she produced positive emotional expressions and utterances (Fugazza et al. 2018). Like in our study, the duration of exposure to the novel object was two minutes. The puppies were even more likely to gaze alternate when the social partner was the human experimenter than when either with their neutral mother (88.8%) or a neutral unfamiliar dog (80%), confirming domestic dogs’ strong predisposition to making eye contact with humans from a young age. Puppies’ gaze alternations in Fugazza et al. (2018) were clearly functional, as they adjusted their behaviour depending on whether the experimenter reacted positively or neutrally to the novel object (social referencing).

It appears that young puppies are more likely to gaze alternate in situations involving a potentially threatening object than in the unsolvable task paradigm (at least in the same time frame), as also confirmed by the much shorter latencies to the first gaze in the novel object test (median 17.2 s) than in the unsolvable task (median 50.8 s) in the current study. Of course, from an evolutionary viewpoint, it can be highly adaptive to gaze at more experienced individuals when exposed to a potential threat in order to react adequately to this stimulus, so this finding is not surprising. Furthermore, it is to be expected that dogs try to pursue the unsolvable task on their own for a while, having been previously successful.

It is conceivable that puppies’ intentions differ between novel object and unsolvable task paradigms. Looking back during an unsolvable task has been interpreted as a strategy to achieve a goal (Hirschi et al. 2022; reviewed in Prato-Previde and Marshall-Pescini 2014), whereas gazing at humans in face of ambiguity may indicate information-seeking (Graham et al. 2021; Striano and Rochat 2000; Prato-Previde and Marshall-Pescini 2014). Nonetheless, in both subtests, there was a correlation between gaze alternations and whimpering of similar magnitude, which would be consistent with a communicative function of the observed gaze alternations. Moreover, despite likely different underlying motivations in the two subtests, the frequency of gaze alternations in the two contexts was significantly correlated in the current study. Thus, dog puppies show individual differences in human-directed gazing that is consistent across contexts.

To our knowledge, only one other study to date reported on a possible association between (adult) dogs’ gazing across different contexts, an unsolvable task and a social referencing task (gazing at a human’s face following the disruption of a dyadic social game). An exploratory factor analysis over 15 variables from a cognitive test battery revealed that these two behaviours loaded together on a single component (MacLean et al. 2017). Thus, our data strengthen the notion that propensity for human-directed gazing is a consistent individual trait in domestic dogs and that individual differences in this characteristic emerge early in ontogeny.

A genetic basis for gazing at humans during unsolvable tasks has been identified (Hori et al. 2013; Persson et al. 2018). Moreover, there is some evidence that individual differences in human-directed gazing are associated with differences in sociability (Jakovcevic et al. 2012) and anxiety (Passalacqua et al. 2013). Future studies should investigate further associations of gazing with personality traits and whether such individual differences in gazing behaviour remain stable across development. This is of special interest because one study found an association of gazing during an unsolvable task with detection dog success: dogs that gazed longer at the experimenter when tested at 11 months were more likely to qualify for service at 12 months (Lazarowski et al. 2019).

## Limitations and future directions

Producing gaze alternations between an object of interest and another individual is often interpreted as referential communication (e.g. Carpenter et al. 1998; Miklósi et al. 2000; McElligott et al. 2020; Nawroth et al. 2016; Savalli et al. 2014), with other attention-getting behaviours such as vocalisations reinforcing the notion of intentionality (e.g. Miklósi et al. 2000; Marshall-Pescini et al. 2013; Savalli et al. 2014). Therefore, in the current study, the performance of gaze alternations between a person and the objects of interest and the correlations with whimpering in both contexts indicate a communicative function of puppies’ gazing. Nonetheless, other explanations for the observed gaze alternations, such as checking/monitoring behaviour or momentary shifts of attention elicited by the environment (Dezecache et al. 2019; Graham et al. 2021; Malavasi and Huber 2016; Tomasello et al. 2005) cannot be ruled out completely based on the experimental design. Follow-up studies should thus explore additional markers of intentionality, including persistence and elaboration when the person present is unresponsive, as well as include control conditions with humans inattentive or absent, and with no objects of interest present (cf. Gaunet and Deputte 2011; Graham et al. 2020; Marshall-Pescini et al. 2013; Mocha and Burkart 2021).

The comparison of our results with those of previous studies strongly suggests the importance of extensive human socialisation for gaze alternations to emerge. For drawing firm conclusions, future studies should systematically test puppies with different levels of human socialisation using the same methodology for all subjects. Likewise, it is possible that breed differences may emerge at an early age when larger sample sizes of puppies from different breed groups are tested in a standardised manner.

Since to our knowledge gaze alternations have never been studied in puppies younger than those aged 41 days in the current study, it is possible that gaze alternations can be shown at an even earlier age than reported here. This could be addressed by longitudinal or cross-sectional studies.

## Conclusions

Our findings suggest that dogs have a genetic preparedness to communicate with humans via gazing very early in ontogeny. In well-socialised dogs, gaze alternations are shown from a very young age, similar as in human children and unlike in great apes and wolves (even when extensively socialised to humans). Comparisons with data from previous studies on the emergence of human-directed gazing suggest that dogs need close contact with humans for gaze alternations to emerge, highlighting the interactive effects of domestication and environmental factors on behavioural development in dogs. The frequency of puppies’ gaze alternations was significantly correlated between subtests, indicating an underlying propensity for gazing at humans despite likely different motivations underlying gazing in the two contexts. Significant correlations of gaze alternations with the duration of whimpering give further evidence for the social-communicative nature of gazing in young dog puppies.

## Electronic supplementary material

Below is the link to the electronic supplementary material.

Supplementary Material 1

## Data Availability

Raw data and the corresponding R script are available from https://osf.io/sqb2n/?view_only=16f1d8bb8ac449689e68828f81c7d029.
