# Supplementary material for: Evidence for the communicative function of human-directed gazing in 6- to 7-week-old dog puppies
Source: Anim Cogn. 2024 Sep 23;27(1):61. doi: 10.1007/s10071-024-01898-y (PMC11420273; doi:10.1007/s10071-024-01898-y)
Supplement: Supplementary file 1 — Supplementary Material 1 [file 10071_2024_1898_MOESM1_ESM.docx]

**Evidence for the communicative function of human-directed gazing in 6 to 7-week-old dog puppies – Supplementary material**

Stefanie Riemer^1,2^*, Alina Bonorand^2^, Lisa Stolzlechner^3^

^1^ Messerli Research Institute, Department of Interdisciplinary Life Sciences, Vetmeduni Vienna, 1210 Vienna, Austria

^2^ Division of Animal Welfare, Vetsuisse Faculty, University of Bern, 3012 Bern, Switzerland

^3^ Department of Cognitive Biology, University of Vienna, 1030 Vienna, Austria

*Correspondence: [riemer.stefanie@gmail.com](mailto:riemer.stefanie@gmail.com) (ORCID: 0000-0001-8008-5291)

**Supplementary Table 1.** Demographic details of the subjects.

| **ID** | **Sex** | **Breed** | **Litter** | **Treatment group from Stolzlechner et al. (2022)*** | **Age at testing (days)** |
| --- | --- | --- | --- | --- | --- |
| Sheltie1_1 | female | Shetland Sheepdog | Sheltie | training | 51 |
| Sheltie1_2 | male | Shetland Sheepdog | Sheltie | control | 51 |
| Sheltie1_3 | male | Shetland Sheepdog | Sheltie | training | 51 |
| Sheltie1_4 | male | Shetland Sheepdog | Sheltie | training | 51 |
| Sheltie1_5 | male | Shetland Sheepdog | Sheltie | control | 51 |
| Sheltie2_1 | female | Shetland Sheepdog | Sheltie | control | 43 |
| Sheltie2_2 | female | Shetland Sheepdog | Sheltie | control | 43 |
| Sheltie2_3 | male | Shetland Sheepdog | Sheltie | training | 43 |
| Sheltie2_4 | female | Shetland Sheepdog | Sheltie | training | 43 |
| Aussie1_1 | male | Australian Shepherd | Aussie1 | control | 46 |
| Aussie1_2 | female | Australian Shepherd | Aussie1 | training | 46 |
| Aussie1_3 | male | Australian Shepherd | Aussie1 | training | 46 |
| Aussie1_4 | female | Australian Shepherd | Aussie1 | control | 46 |
| Aussie1_5 | female | Australian Shepherd | Aussie1 | control | 46 |
| Aussie1_6 | male | Australian Shepherd | Aussie1 | control | 46 |
| Aussie1_7 | female | Australian Shepherd | Aussie1 | training | 46 |
| Aussie1_8 | male | Australian Shepherd | Aussie1 | training | 46 |
| Herder_1 | female | Dutch Shepherd (Hollandse Herder) | Herder | control | 41 |
| Herder_2 | female | Dutch Shepherd (Hollandse Herder) | Herder | control | 41 |
| Herder_3 | female | Dutch Shepherd (Hollandse Herder) | Herder | training | 41 |
| Herder_4 | male | Dutch Shepherd (Hollandse Herder) | Herder | training | 41 |
| Herder_5 | male | Dutch Shepherd (Hollandse Herder) | Herder | training | 41 |
| Herder_6 | female | Dutch Shepherd (Hollandse Herder) | Herder | control | 41 |
| Herder_7 | female | Dutch Shepherd (Hollandse Herder) | Herder | training | 41 |
| Herder_8 | male | Dutch Shepherd (Hollandse Herder) | Herder | control | 41 |
| Aussie2_1 | female | Australian Shepherd | Aussie2 | control | 40 |
| Aussie2_2 | female | Australian Shepherd | Aussie2 | training | 40 |
| Aussie2_3 | female | Australian Shepherd | Aussie2 | training | 40 |
| Aussie2_4 | male | Australian Shepherd | Aussie2 | control | 40 |
| Aussie3_1 | female | Australian Shepherd | Aussie3 | training | 48 |
| Aussie3_2 | female | Australian Shepherd | Aussie3 | control | 48 |
| Aussie3_3 | male | Australian Shepherd | Aussie3 | training | 48 |
| Aussie3_4 | male | Australian Shepherd | Aussie3 | control | 48 |
| Aussie3_5 | female | Australian Shepherd | Aussie3 | control | 48 |
| Aussie3_6 | male | Australian Shepherd | Aussie3 | control | 48 |
| Aussie3_7 | male | Australian Shepherd | Aussie3 | training | 48 |
| Pitbull_1 | female | Pitbull | Pitbull | training | 41 |
| Pitbull_2 | male | Pitbull | Pitbull | training | 41 |
| Pitbull_3 | male | Pitbull | Pitbull | training | 41 |
| Pitbull_4 | female | Pitbull | Pitbull | control | 41 |
| Pitbull_5 | male | Pitbull | Pitbull | control | 41 |
| Pitbull_6 | male | Pitbull | Pitbull | control | 41 |
| Pitbull_7 | female | Pitbull | Pitbull | control | 41 |
| Pitbull_8 | female | Pitbull | Pitbull | control | 41 |
| Pitbull_9 | male | Pitbull | Pitbull | control | 41 |
| Pitbull_10 | male | Pitbull | Pitbull | training | 41 |
| Pitbull_11 | female | Pitbull | Pitbull | training | 41 |
| MiniAussie_1 | male | MiniAussie | MiniAussie | control | 41 |
| MiniAussie_2 | female | Mini Australian Shepherd | MiniAussie | training | 41 |
| MiniAussie_3 | female | Mini Australian Shepherd | MiniAussie | control | 41 |
| MiniAussie_4 | male | Mini Australian Shepherd | MiniAussie | control | 41 |
| MiniAussie_5 | female | Mini Australian Shepherd | MiniAussie | training | 41 |
| MiniAussie_6 | female | Mini Australian Shepherd | MiniAussie | control | 41 |
| MiniAussie_7 | male | Mini Australian Shepherd | MiniAussie | training | 41 |
| Icelandic_1_ | female | Icelandic Sheepdog | Icelandic | training | 42 |
| Icelandic_2 | male | Icelandic Sheepdog | Icelandic | control | 42 |
| Icelandic_3 | female | Icelandic Sheepdog | Icelandic | control | 42 |
| Icelandic_4 | male | Icelandic Sheepdog | Icelandic | training | 42 |
| Icelandic_5 | male | Icelandic Sheepdog | Icelandic | training | 42 |
| Icelandic_6 | female | Icelandic Sheepdog | Icelandic | control | 42 |
| Labrador_1 | female | Labrador retriever | Labrador | control | 41 |
| Labrador_2 | male | Labrador retriever | Labrador | training | 41 |
| Labrador_3 | male | Labrador retriever | Labrador | control | 41 |
| Labrador_4 | male | Labrador retriever | Labrador | control | 41 |
| Labrador_5 | female | Labrador retriever | Labrador | training | 41 |
| Labrador_6 | female | Labrador retriever | Labrador | training | 41 |
| Labrador_7 | male | Labrador retriever | Labrador | training | 41 |
| Labrador_8 | female | Labrador retriever | Labrador | control | 41 |
| Labrador_9 | female | Labrador retriever | Labrador | training | 41 |
| Aussie4_1 | female | Australian Shepherd | Aussie4 | control | 46 |
| Aussie4_2 | female | Australian Shepherd | Aussie4 | control | 46 |
| Aussie4_3 | female | Australian Shepherd | Aussie4 | control | 46 |
| Aussie4_4 | female | Australian Shepherd | Aussie4 | training | 46 |
| Aussie4_5 | female | Australian Shepherd | Aussie4 | training | 46 |
| Aussie4_6 | female | Australian Shepherd | Aussie4 | training | 46 |
| Aussie4_7 | female | Australian Shepherd | Aussie4 | training | 46 |
| Setter_1 | female | English Setter | Setter | training | 43 |
| Setter_2 | male | English Setter | Setter | control | 43 |
| Setter_3 | female | English Setter | Setter | training | 43 |
| Setter_4 | male | English Setter | Setter | training | 43 |
| Setter_5 | female | English Setter | Setter | control | 43 |
| Setter_6 | female | English Setter | Setter | control | 43 |
| Setter_7 | female | English Setter | Setter | training | 43 |

***** relevant for Stolzlechner, L., Bonorand, A., & Riemer, S. (2022). Optimising puppy socialisation–short-and long-term effects of a training programme during the early socialisation period. *Animals*, *12*(22), 3067. There were no significant differences between treatment groups in the current study.

**Supplementary Table 2**. Cronbach’s alpha as a measure of inter-rater reliability.

| **Variables** | **Cronbach’s alpha** |
| --- | --- |
| Novel object – duration of whimpering | 0.99 |
| Novel object – frequency of gaze alternations | 0.82 |
| Unsolvable task – duration of whimpering | 0.83 |
| Unsolvable task – frequency of gaze alternations | 0.91 |
